# HIV prevalence and correlated factors among male clients of female sex workers in a border region of China

**DOI:** 10.1371/journal.pone.0225072

**Published:** 2019-11-07

**Authors:** Jing Zhu, Dan Hu, Yueqi Yin, Zhibin Zhu, Ning Wang, Bei Wang

**Affiliations:** 1 Key Laboratory of Environmental Medicine and Engineering, Ministry of Education, Department of Epidemiology and Medical Statistics, School of Public Health, Southeast University, Nanjing, Jiangsu, P.R. China; 2 Department of Health Policy and Management, Nanjing Medical University, Nanjing, Jiangsu, P.R. China; 3 Hekou Center for Disease Control and Prevention, Hekou, Yunnan, P.R. China; 4 National Center for AIDS/STD Control and Prevention, Chinese Center for Disease Control and Prevention, Beijing, P.R. China; Sefako Makgatho Health Sciences University, SOUTH AFRICA

## Abstract

**Objectives:**

To assess the prevalence of human immunodeficiency virus (HIV) infection and the correlated risk factors among male clients of female sex workers (FSWs) in a Chinese–Vietnamese border region in Yunnan Province, China.

**Methods:**

A cross-sectional survey was conducted between 2014 and 2015 in Hekou County, Yunnan Province, China. Convenience sampling and snowball sampling methods were used to recruit male clients for a questionnaire survey to collect information on demographics, sexual behavior, and drug use. Blood and urine samples were collected for testing of HIV/sexually transmitted infections (STIs) and drug use. Multivariate logistic regression was used to examine factors correlated with HIV infection.

**Results:**

Of 776 respondents who participated in the study, 721 (92.91%) were Chinese and 55 (7.09%) were Vietnamese. Overall HIV prevalence in male clients of FSWs was 2.06%, 128 (16.49%) were infected with HSV-2, and five (0.64%) tested syphilis-positive. Two-thirds (68.81%) of respondents reported always using condoms with FSWs, and 89.05% reported condom use in the last episode of commercial sex. Male clients from Vietnam were significantly more likely to take morphine (9.09%) compared with Chinese male clients of FSWs. Age ≥50 years (OR: 8.11, 95%*CI*: 1.26–52.16) and morphine positivity (OR: 7.35, 95%*CI*: 1.42–38.06) were associated with HIV infection in the multivariate logistic regression model.

**Conclusion:**

The relatively high proportion of male clients of FSWs who have numerous sexual partners and use condoms less frequently make them serve as important bridges for HIV transmission from FSWs to the low-risk general population. The positive association between morphine positivity and HIV infection confirmed illegal drug use as another important route for acquiring HIV infection in addition to sexual transmission, indicating that innovative interventions addressing both drug use and risky sexual behaviors are greatly required for male clients. Respondents aged ≥50 years have a higher risk of HIV infection, which emphasizes that older male clients of FSWs should be focused in future HIV prevention interventions in the border regions of China.

## Introduction

Since 2007, sexual transmission has replaced intravenous drug use as the primary mode of human immunodeficiency virus (HIV) transmission in China [1–2]. During the first half of 2016, it was estimated that 66.3% of novel HIV infections in China were acquired through unprotected heterosexual intercourse, which was considerably higher than the 2007 estimate of 37.9% [3–5]. Owing to the increasing role of commercial sexual transmission the HIV epidemic, female sex workers (FSWs) as well as their male clients are considered high-risk populations for HIV infection[6,7]. Pan S *et al*. [8] concluded that approximately 6%–9% of male adults aged between 18 and 60 years have ever engaged in commercial sexual behaviors with FSWs in China. In 2012, McLaughlin *et al*. [9] estimated through a meta-analysis that HIV prevalence among Chinese male clients of FSWs was 0.68%, which is more than ten times higher than the estimate for adults in the general population. In addition, the HIV prevalence estimate among male clients in southwest China is significantly higher than among those in other regions (2.54% vs. 0.24%, *P* = 0.001).

Yunnan Province in the southwest is one of the epicenters of the HIV epidemic in China[10]. Due to its special geographic location near the “Golden Triangle,” the border regions of Myanmar, Thailand, and China, HIV in this region has primarily spread through injecting drug use[1,11]. However, the role of sexual transmission has increased, and since 2005, most novel HIV infections were acquired through sexual contact [12]. Hekou County, with a population of 103,400, is the national border port of Yunnan Province and shares a 193 kilometer land border with Lao Cai Province in the north part of Vietnam [13]. Along with the rapid development of economic cooperation between China and Vietnam, a growing number of Vietnamese women have married or sold sex in China. It has been estimated that about 700 FSWs operate in Hekou county, including 600 Vietnamese and 100 Chinese[14]. Most Vietnamese FSWs gather in brothel-based sexual markets near the border crossing in Hekou named “Vietnamese Street”; in contrast, Chinese FSWs are often scattered in entertainment venues and hotels all over the Hekou County. The flourishing commercial sex industry has also attracted large numbers of male clients to Hekou. Previous research has indicated that HIV prevalence of FSWs in Hekou County declined significantly compared with past reports[15]. However, little is known about the HIV prevalence and the associated risks among male clients of FSWs in recent years.

This study focuses on HIV prevalence and attempts to identify the factors associated with HIV infection of male clients of FSWs. The study aimed to provide suggestions for HIV prevention interventions that target male clients of FSWs in border regions.

## Methods

This cross-sectional research for male clients of FSWs was conducted every 5 months from June 2014 through December 2015 in Hekou County, Honghe Prefecture, Yunnan Province, China. The investigation lasted one month in each time during the research. Men were considered eligible for participation if they were at least 16 years old, self-reported having paid for commercial sexual services in the past 12 months, willing to answer our study questions on demographics, sexual and drug using behaviors, and provide written voluntary informed consent. By Chinese law, participants who were between 16–18 years were considered as having full capacity for civil conduct if they relied on their own labor income [14]. Therefore, in our study, male clients of FSWs under age 18 signed the same written voluntary informed consent as participants older than age 18, which was also approved by the Ethical Review Committee of China CDC.

Male participants were recruited from brothel-based commercial sex markets and entertainment venues near the border crossing in Hekou County. The local outreach workers and health officials of Hekou center for disease control and prevention (CDC) approached male clients of FSWs entering these commercial sex venues to inform them about the study. In addition, snowball sampling was used to recruit potential male clients through the acquaintances of FSWs, FSWs’ employers and other male clients. FSWs, FSWs’ employers and recruited male clients can provide multiple referrals. Each new referral can provides with more data for referral and so on. The study provided an incentive of 10 CNY (1.5 USD) to the referring person for each participant recruited. Each male client in this study received a unique personal identity number associated with fingerprint of the right index finger to avoid duplicate participation.

Bilingual outreach workers and health officials of Hekou CDC collected data on demographic characteristics, sexual behavior, and drug use through face-to-face interview by using a structured questionnaire. All the study staff were trained in standardized methods of data collection by specialists from the National Center for AIDS/STD Control and Prevention (NCAIDS) of China CDC. Graduate students from Southeast University and NCAIDS of China CDC were present for quality assurance of the interview and questionnaire. Venous blood and urine samples were collected by trained physicians of Hekou County People’s Hospital. All the participants were compensated 50 CNY (7 USD) for participation in this study.

Specimens were collected as follows: 7 ml venous blood for diagnosis of HIV, herpes simplex virus type 2 (HSV-2), and syphilis, and 15 ml urine to test for morphine, ketamine, and amphetamine. Plasma specimens were cold transported to the laboratory and screened for HIV antibodies by using enzyme-linked immunosorbent assay (ELISA; Organon Teknika,Co., Ltd, Boxtel, the Netherlands), and positive tests were confirmed by HIV-1/2 Western blot assay (HIV Blot 2.2 WBH; Genelabs Diagnostics, Singapore). Blood specimens were also tested for antibodies toHSV-2 by ELISA(HerpeSelect-2 ELISA IgG; Focus Technologies, Cypress, CA, USA). Syphilis was tested for by using the syphilis rapid plasma regain (RPR Diagnostics kit; Shanghai Kehua, China) for *Treponema pallidum*, and positive specimens were confirmed by using a *T*. *pallidum* particle assay (Serodia-TPPA; Fujirebio Inc.,Fuji, Japan). Urine samples were tested for illegal drug use by using morphine, ketamine, and amphetamine gold conjugate test strips (ABON; Hangzhou, China).

Data from the research questionnaire and laboratory tests were entered into Epidata 3.1 (Odense, Denmark) and analyzed by using SPSS 22.0 (IBM Inc., Armonk, USA). Medians and interquartile range (IQR) values were used to describe non-normal continuous data; frequencies and percentages were calculated for each level of categorical variables. Chi-squared, Fisher’s exact tests, and Wilcoxon non-parametric tests were used to examine the differences and associations in univariate analyses. Logistic regression was used to examine the correlations between HIV infection and its cofactors. For univariate analyses, continuous data were transformed into categorical data based on their cumulative percent distribution. Variables significant at *P* < 0.2 in univariate analyses were included in the multivariate model. Variables were entered and eliminated from the model in a stepwise manner, with entry *P* < 0.1and exit *P* > 0.05. All of the statistical tests were two sided, with significance determined at *P* < 0.05.

This study received approval from the Ethical Review Committee of China CDC.

## Results

A total of 776 male clients of FSWs were approached to participate in our research. All the male respondents who met the inclusion criteria finished the interview questionnaire and collection of biological specimens. Male participants consisted of 721 (92.91%)Chinese and 55 (7.09%) Vietnamese subjects. For Chinese respondents, 202 (26.03%) of whom were residents of Hekou county, 519 (66.88%) were from other provinces of China. Vietnamese male clients in the study were all from Vietnam. Among 776 male clients of FSWs, the median age was 36 years (IQR: 27–45 years), and 67.27% (522/776) were married or cohabiting. Four-fifths of participants had less than nine years of school (n = 621, 80.03%), 157 (20.23%) had a monthly income more than 5000 Chinese yuan (CNY, 750 USD). Table 1 shows that the demographic variables of age, marital status, and monthly income were significantly different between the three groups of male clients. Respondents from Vietnam were younger and had a higher percentage of singles than their Chinese counterparts.

**Table 1:** Demographic characteristics among male clients of FSWs.

Variable	Residency of male clients of FSWs[n(%)]			*P* value
	Local residence in Hekou county	Other regions of China	Vietnam	
**Age(years)** [M(P25~P75)]	42 (31.75~47.25)	36^a^ (27~44)	25^a^^b^ (21~36)	<0.001
**Marital status**				<0.001
Single	41(20.30)	141(27.17)^a^	31(56.36)^a^^b^	
Married or cohabiting	144(71.29)	354(68.21)	24(43.64)	
Divorced or widowed	17(8.42)	24(4.62)	0	
**Education(years)**				0.088
≤6	70(34.65)	173(33.33)	15(27.27)	
7–9	96(47.52)	246(47.40)	21(38.18)	
≥10	36(17.82)	100(19.27)	19(24.55)	
**Monthly income(CNY)**				<0.001
<3000	141(69.80)	159(30.64)^a^	32(58.18)^a^^b^	
3000~5000	46(22.77)	220(42.39)	21(38.18)	
>5000	15(7.43)	140(26.97)	2(3.64)	

^a:^ Compared with “Local residence in Hekou county”, *P*<0.05

^b:^ Compared with “Other regions of China”, *P*<0.05.

The median age of sexual debut of male clients in the study was 20 years (IQR: 18–22 years). Of those who reported currently having a regular sexual partner (75.77%, 588/776), only 10.03% (59/588) reported always using condoms with their regular partner. In addition, 181 (23.32%) male clients had other non-commercial sexual partners (unpaid sexual partners who are not their girlfriends or wives) at the time of interview and of these 46.41% (84/181) of them reported that they never used condoms. In addition, 55.15% (428/776) of male clients chose “Vietnam street” as the most frequent site for visiting FSWs and 14.82% (115/776) would prefer to patronize the same FSWs at each time. Among male respondents, 68.81% (534/776) reported always using condoms with FSWs, and 89.05% (691/776) used condoms in the last episode of commercial sex (Table 2). Compared with Chinese clients of FSWs, Vietnamese participants had a younger age of sexual debut were more likely to choose “Vietnam street” as the most frequent site for visiting FSWs in Hekou County.

**Table 2:** Sexual behaviors of male clients of FSWs.

Variable	Residency of male clients of FSWs[n(%)]			*P* value
	Local residence in Hekou county	Other regions of China	Vietnam	
**Age of sexual debut** [M(P25~P75)]	20 (18~22)	20 (18~22)	18^a^^b^ (18~20)	0.014
**Have regular sexual partner (girlfriend or wife)**				0.151
Yes	162(80.20)	388(74.76)	38(69.09)	
No	40(19.80)	131(25.24)	17(30.91)	
**Condom use with regular sexual partner (girlfriend or wife, n = 588)**				0.079
Always	10(6.17)	44(11.34)	5(13.16)	
Sometimes	39(24.07)	120(30.93)	9(23.68)	
never	113(69.75)	224(57.73)	24(63.16)	
**Have other non-commercial sexual partners**				0.742
Yes	51(25.25)	117(22.54)	13(23.64)	
No	151(74.75)	402(77.46)	42(76.36)	
**Condom use with other non-commercial sexual partners(n = 181)**				0.562
Always	13(25.49)	38(32.48)	4(30.77)	
Sometimes	13(25.49)	28(23.93)	1(7.69)	
never	25(49.02)	51(43.59)	8(61.54)	
**Most frequent site for visiting FSWs**				0.015
Entertainment venues	25(12.38)	75(14.45)	2(3.64)^a^^b^	
Hotel	27(13.27)	79(15.22)	8(14.55)	
“Vietnam street”	112(55.45)	275(52.99)	41(74.55)	
Others	38(18.81)	99(17.34)	4(7.27)	
**Predilection of FSWs**				0.050
Different at each time	22(10.89)	66(12.72)	13(23.64)^a^^b^	
The same at each time	40(19.80)	67(12.91)	8(14.55)	
Young	126(62.38)	340(65.51)	34(61.82)	
Others	14(6.93)	46(8.86)	0	
**Condom use with FSWs**				0.621
Always	135(66.83)	364(70.13)	35(63.64)	
Sometimes	54(26.73)	121(23.31)	14(25.45)	
never	13(6.44)	34(6.55)	6(10.91)	
**Condom use with the last FSW**				0.048
Yes	171(84.65)	472(90.94)^a^	48(87.27)	
No	31(15.35)	47(9.06)	7(12.73)	

^a:^ Compared with “Local residence in Hekou county”, *P*<0.05

^b:^ Compared with “Other regions of China”, *P*<0.05.

Regarding HIV/STIs and drug using tests, 16 (2.06%) were identified as HIV-positive, 128 (16.49%) were infected with HSV-2, and 5 (0.64%) tested syphilis-positive. Twenty-two male respondents were categorized as illegal drug users by positive urinalysis for morphine. Table 3 illustrates comparisons of HIV, HSV-2, and syphilis status between Chinese and Vietnamese male clients and shows that there were no significant differences. However, participants from Vietnam had a higher rate of morphine positivity compared with the two groups of Chinese participants.

**Table 3:** HIV/STIs and illicit drug test of male clients of FSWs.

Variable	Residency of male clients of FSWs[n(%)]			*P* value
	Local residence in Hekou county	Other regions of China	Vietnam	
**HIV infection**				0.839
Negative	199(98.51)	507(97.69)	54(98.18)	
Positive	3(1.49)	12(2.31)	1(2.06)	
**HSV-2 infection**				0.074
Negative	160(79.21)	438(84.39)	50(90.91)	
Positive	42(20.79)	81(15.61)	5(9.09)	
**Syphilis infection**				0.536
Negative	202(100)	514(99.04)	55(100)	
Positive	0	5(0.96)	0	
**Morphine urine test**				0.015
Negative	197(97.52)	507(97.69)	50(90.91)^a^^b^	
Positive	5(2.48)	12(2.31)	5(9.09)	

^a:^ Compared with “Local residence in Hekou county”, *P*<0.05

^b:^ Compared with “Other regions of China”, *P*<0.05.

Correlated factors for HIV infection are shown in Table 4. Age distribution, marital status, education, predilection of FSWs, the last commercial sexual partner, morphine test, and HSV-2 test were screened considered significant for HIV infection at the univariate level. Age ≥50 years (OR: 8.11, 95%*CI*: 1.26–52.16) and morphine positivity (OR: 7.35, 95%*CI*: 1.42~38.06) were retained in the multivariate logistic regression model as being associated with greater odds of HIV infection.

**Table 4:** Correlated factors of HIV among male clients of FSWs.

Variable	n	n (%) of HIV infections	Univariate OR (95%*CI*)	Multivariate OR (95%*CI*)
**Age distribution**				
<30	249	2(0.80)	1	1
30~	205	4(1.95)	2.46(0.45~13.56)	1.99(0.30~13.20)
40~	214	2(0.93)	1.17(0.16~8.34)	1.03(0.12~9.04)
50~	108	8(7.41)	9.88(2.06~47.34)‡	8.11(1.26~52.16)‡
**Marital status**				
Single	213	2(0.94)	1	
Married or cohabiting	522	10(1.92)	2.06(0.45~9.48)	
Divorced or widowed	41	4(9.76)	11.41(2.02~64.52)‡	
**Education**				
≤6	258	9(3.49)	1	
7–9	363	6(1.65)	0.47(0.16~1.32)§	
≥10	155	1(0.65)	0.18(0.02~1.43)§	
**Predilection of FSWs**				
Different at each time	101	1(0.99)	1	
The same at each time	115	3(2.61)	2.68(0.27~26.17)	
Young	500	7(1.40)	1.42(0.17~11.67)	
Others	60	5(8.33)	9.09(1.04~79.79) †	
**The last commercial sexual partner**				
Chinese FSW	185	1(0.54)	1	
Vietnamese FSW	591	15(2.54)	4.79(0.63~36.52)§	
**Morphine test**				
Negative	754	14(1.86)	1	1
Positive	22	2(9.09)	5.29(1.13~24.82) †	7.35(1.42~38.06)†
**HSV-2 test**				
Negative	648	10(1.54)	1	
Positive	128	6(4.69)	3.14(1.12~8.79)†	

OR: odds ratio; CI: confidence interval; FSW: female sex worker; HIV: HIV: human immunodeficiency virus; HSV-2 herpes simplex virus type 2.

§P<0.20

†P<0.05

‡P<0.01.

## Discussion

The HIV prevalence rate of male clients in this study was 2.06%, which is lower than the estimates of Xia Jin, *et al*. [16] and JJ Xu *et al*. [17] in other regions of Honghe Prefecture, Yunnan Province, China, and also lower than the estimate for male clients in another border region of north Vietnam by NT Nguyen *et al*. [18]. KH Reilly *et al*. [19] reported that HIV prevalence of male clients of FSWs in Hekou County in 2010 was as high as 9.2%. Compared with past studies, the HIV prevalence of male clients in this study has declined significantly. Previous research indicates that the HIV prevalence of FSWs in Hekou County showed a continuous decline in recent years due to the effect of health education and behavioral interventions targeting FSWs [15]. The relatively low HIV prevalence of male clients in this study may be attributed to the prevention and interventions for commercial sexual transmission of HIV that are provided by Hekou CDC. In addition, the change in proportions of high-risk populations and patterns of risk behavior in past years among male clients maybe important factors associated with the reduction in HIV infection.

The results of demographic questions showed that age and marital status among the three groups of male clients were statistically different. Vietnamese participants were younger than Chinese respondents. The proportion who report being married or cohabiting was significantly lower in Vietnamese clients of FSWs compared with Chinese clients, either those from Hekou country or from other regions. This indicates that Chinese male clients may play a greater role than Vietnamese male clients in transmitting HIV to regular, unpaid sexual partners.

The observation that 55.15% of male clients chose “Vietnam street” as the most frequent site for commercial sex indicates that male clients in Hekou County are more likely to visit Vietnamese FSWs and suggests that behaviors that pose an HIV risk are more likely to occur in these groups. Most male clients (68.81%) reported always using condoms in commercial sex, which is a significant improvement over the estimate for male clients in Hekou in 2010[19] and also higher than the estimate from past studies of male clients in other regions of Yunnan Province, China [16,17,20]. However, 75.77% of male respondents reported that they currently have a regular sexual partner, of whom only 10.03% regularly use condoms; in addition, 23.32% of male clients reported currently having other non-commercial sexual partners, of whom nearly 50% reported never having used condoms. Previous research suggests that males in Asian countries always have dominance when it comes to deciding on condom use [14,18,21]. Therefore, further research that concentrates on promoting condom use among male clients is important in preventing and controlling the spread of HIV by sexual transmission across the China–Vietnam border.

In this study, there were no statistically significant differences in the prevalence of HIV/STIs among the three groups of participants, which suggests that this risk is similar among Chinese and Vietnamese male clients. However, respondents from Vietnam had a significantly higher rate of morphine positivity, which suggests a greater risk of HIV infection through drug using behaviors that will require attention in future HIV prevention interventions[22,23].

Through multivariate logistic regression, morphine positivity (OR: 7.35, 95%*CI*: 1.42–38.06) and age ≥50 years (OR: 8.11, 95%*CI*: 1.26–52.16) were indicated as significant risk factors for HIV infection. Drug use has been found to be associated with HIV infection in many previous studies [24–26]. Xia Jin*et al*. [16] estimated that HIV prevalence of male clients of FSWs in Kaiyuan City, Yunnan Province, China, was 6.0%; among drug users, it was as high as 30.8%. In our study, morphine positivity was associated with a 7.35-fold increased risk in HIV prevalence, which confirms that drug use remains a salient risk factor for HIV infection in southwestern China. In addition, one possible explanation as to why male clients aged ≥50 years had higher risk of acquiring HIV is that these clients may have a longer history of exposure through unprotected sexual behaviors, drug use, and other risks for HIV. Chen Y *et al*. [27] found that older male clients were more vulnerable to HIV infection because of their low level of education and HIV-related knowledge and even lesser condom use in GuangXi Province, China. Therefore, it is a great challenge to provide effective HIV interventions for older male clients of FSWs especially in the border regions of China. Furthermore, some previous studies found that lack of regular sexual partner and low monthly income were associated with HIV infection of male clients of FSWs but not significantly in this study [16, 21]. Different regions may be important reason.

## Limitation

This study faces several limitations. First, because commercial sex work is illegal in China, it is quite difficult to obtain a sampling frame and take a random selection of participants. Due to our recruitment methods, male participants in our study may be more accessible and willing to participate in our research. Actually, a lot of male clients of FSWs refused participating in the study when the outreach workers approached them. This potential selection bias suggests that participants may not be representative of all the male clients in Hekou County or other border regions of China and may have lower risk for HIV. Second, as taboo and sensitive issues were included in this study and questionnaire interviews were mainly conducted by staff and health officials of Hekou CDC, male participants may be subject to desirability bias. In addition, recall bias among male clients may also affect the correlation between HIV infection and its cofactors. Moreover, the cross-sectional design of our study means that it can only provide information on correlations and cannot determine the causal relationship between HIV infection and its cofactors.

## Conclusion

Male clients of FSWs in the border region are not only vulnerable for HIV but also play a considerable role in transmitting HIV across the border to the general, lower risk population through multiple sexual partners, low or inconsistent condom use, and other HIV risk behaviors[16]. Gaining a better understanding of the factors correlated with HIV infection in male clients of FSWs is important for designing effective interventions. Considering that male clients of FSWs are migrant and hidden populations with high HIV risk[28], broad-based HIV-related health education should be considered to inform the potential male clients in the general population[29]. Further studies on innovative interventions that addresses both drug use and sexual risk behavior and targets male clients are greatly required for HIV control and prevention in the border region[19,30].

## Supporting information

S1 Appendix Data set used for the analyses of male clients of FSWs. (SAV)

S2 Appendix Ethical review. (PDF)

S3 Appendix Ethical review translation. (DOCX)

S4 Appendix Questionnaire for male clients (Chinese). (DOC)

S5 Appendix Questionnaire for male clients (English). (DOC)
