# Supplementary material for: HIV prevalence and correlated factors among male clients of female sex workers in a border region of China
Source: PLoS One. 2019 Nov 7;14(11):e0225072. doi: 10.1371/journal.pone.0225072 (PMC6837524; doi:10.1371/journal.pone.0225072)
Supplement: S2 Appendix — (PDF) [file pone.0225072.s002.pdf]

中国疾病预防控制中心
性病艾滋病预防控制中心

中国疾病预防控制中心
性病艾滋病预防控制中心伦理委员会
项目评审报告

项目编号：X120331209

经中国疾病预防控制中心性病艾滋病预防控制中心伦理委员会专家评审后，认为下列项目符合我国伦理学方面的要求，项目可以进行实施。

项目名称：　我国艾滋病流行趋势、疫情评估和预测数学模型研究

项目负责人：汪宁　教授

单　　位：　中国疾病预防控制中心性病艾滋病预防控制中心

评审日期：　2012 年 3 月 31 日

批准日期：　2012 年 3 月 31 日

主席：(签字) 王若涛

王若涛

中国疾病预防控制中心

性病艾滋病预防控制中心伦理委员会

地址：北京市昌平区昌百路155号　邮编：102206　电话：58900922　传真：58900920　E-mail：office606@chinaaids.cn
