# Supplementary material for: HIV prevalence and correlated factors among male clients of female sex workers in a border region of China
Source: PLoS One. 2019 Nov 7;14(11):e0225072. doi: 10.1371/journal.pone.0225072 (PMC6837524; doi:10.1371/journal.pone.0225072)
Supplement: S3 Appendix — (DOCX) [file pone.0225072.s003.docx]

Chinese Center for Disease Control and Prevention

National Center for AIDS/STD Control and Prevention

Chinese Center for Disease Control and Prevention

The Ethical Review Committee of National Center for AIDS/STD Control and Prevention

Project Review Report

Project Number: X120331209

The Ethical Review Committee of National Center for HIV/AIDS Control and Prevention, Chinese Center for Disease Control and Prevention has reviewed the Project. It is recognized that the right and the welfare of the subject are adequately protected. The Project conforms to the ethical requirement and can be implemented.

Title of Project: Research on epidemic trend, assessment and prediction mathematical method of AIDS

Principal Investigator of the Project: Ning Wang

Institute: Chinese Center for Disease Control and Prevention

Department/Division: National Center for AIDS/STD Control and Prevention

Date Submitted: March 31, 2012

Date Approved: March 31, 2012

Signature: Ruotao Wang

Ruotao Wang

Chair, NCAIDS, China CDC Ethical Review Committee
