# Supplementary material for: HIV prevalence and correlated factors among male clients of female sex workers in a border region of China
Source: PLoS One. 2019 Nov 7;14(11):e0225072. doi: 10.1371/journal.pone.0225072 (PMC6837524; doi:10.1371/journal.pone.0225072)
Supplement: S4 Appendix — (DOC) [file pone.0225072.s004.doc]

**男性健康行为调查问卷**

PID号：**□□□□□□**

您好，中国疾病预防控制中心正在进行一个调查，想了解大家对健康和疾病问题的认识。您对任何一个问题的回答都是保密的，您的姓名不会出现在调查表上。如果您有什么问题，请随时提出。您对这些问题的如实回答，将帮助我们更好地了解人们对这些问题的看法和做法。谢谢您的合作。

填写说明：请在“[__]”内填写数字或者选项代码或者划“X”，可多选选项请在相应答案前“[__]”内划“X”，“___”上请填写中文。

调查员____________日期：____________； 审核员_____________日期_____________

**第一部分 基本情况**

A101.出生年/月 [__|__|__|__]年 [__|__]月

A102. [__]婚姻状况 1)未婚 2)在婚 3)同居 4)离婚或丧偶

A103.[__]户籍

1)本县 2)本州其它县市 3)本省其它州 4)外省（_______省） 5)外籍（______国）***🡺跳至A104b题***

A104a. [__]如果是中国人,请回答民族 ***🡺回答该问题后跳至105题***

1)汉族 2)回族 3)哈尼族 4)彝族 5)其它__________

A104b.[__]如果是越南人,请回答民族

1)京族 2)占族 3)华族 4)傣族 5)苗族 6)孟族 7)高棉族 8)其它__________

A105.职业 1)商人 2)工人 3)司机 4)农民 5)其他____________________

A106.[__]文化程度 1)文盲 2)小学 3)初中 4)高中或中专 5)大专及以上

A107.[__]目前家庭住处 1)农村 2)县城/乡镇 3)中小城市 4)大城市(直辖市、省会城市)

A108.[__]你听懂越南语吗？ 1）都能听懂 2）能听懂一部分 3）一点也听不懂

A109.[__]你会说越南语吗？ 1）说的很好 2）会说一些 3）一点也不会说

**第二部分 工作和生活情况**

B201.你来河口的原因是什么？(可多选)

[__]B201 1)生意原因 [__]B201 2)家庭需要 [__]B201 3)工作调动

[__]B201 4)旅游 [__]B201 5)本地居民 [__]B201 6)其它__________

B202.过去5年，你换了几次住所(跨城市或乡镇)？ [__|__|__]次 ***(如果填0🡺跳至B204题)***

B203.你更换住所的原因是什么？***(可多选，请在选项中划X)***

B203a). [__]生意原因 B203b) .[__]家庭需要 B203c) .[__]工作调动

B203d).[__]经济原因，为了赚钱生存 B203e) .[__]其它(请注明)__________________

B204.[__]目前每月收入（元） 1)收入是0，用自己或家人的积蓄，退休金等

2)1～ 3)500～ 4)1000～ 5)2000～ 6)3000～ 7)5000～

B205.[__]家庭月收入（元） 1)收入是0，用自己或家人的积蓄，退休金等

2)1～ 3)1000～ 4)2000～ 5)4000～ 6)6000～ 7)8000～

B206.[__]目前子女数 1) 无 2) 1个 3) 2个 4) 3个 5) 3个以上

B207.[__]你喝酒吗？ 1)是 2)否

B208.[__]你吸烟吗? 1)吸 2)不吸

**第三部分 医疗情况**

C301.你是否有过下列经历？ 1)是 2)否 3)不知道

C301a). [__] 拔牙、补牙或洗牙 C301b). [__] 和别人共用剃刀

C301c). [__] 接受输血或其他血制品 C301d). [__] 献血

C301e). [__] 纹身 C301f). [__] 割礼(包皮切割术)

C301g). [__] 手术(不包括割礼) C301h). [__] 与别人共用牙刷

C302.过去12个月中，是否有过以下症状？***(可多选)***  1)是 2)否 3)不知道

C302a). [__] 生殖器周围瘙痒 C302b) [__] 排尿疼痛或烧灼感

C302c) [__] 尿道口流脓或有分泌物 C302d) [__] 生殖器有增生物或溃疡

C302e) [__] 大腿根部淋巴结肿大 C302f) [__] 没有任何症状***🡺跳至CD401题***

C303. 现在是否还有这些症状？ 1)是 2)否

C303a) [__]生殖器周围瘙痒 C303b) [__]排尿疼痛或烧灼感

C303c) [__]性尿道口流脓或有分泌物 C303d) [__]生殖器有增生物或溃疡

C303e*)* [__]大腿根部淋巴结肿大

**第四部分 性病/艾滋病知识和自觉危险性**

D401. [__] 你听说过性病吗? 1)是 2)否***🡺跳至D405a题***

D402. [__] 你认为你感染性病的危险性有多大？

1)没有危险 2)低危险 3)中危险 4)高危险 5)不知道

D403. [___] 最近一年，你是否曾被诊断患过性病？ 1)是 2)否***🡺跳至D405a题***

D404. 最近一年，你曾被诊断患过何种性病？(可多选)

[__]D404a).淋病; [__]D404b).梅毒; [__]D404c. 尿道沙眼衣原体感染; [__]D404d. 尖锐湿疣

[__]D404e).生殖器疱疹 [__]D404f).其它(请注明)___________________

D405a.[___]一个感染了艾滋病病毒的人能从外表上看出来吗？ ①能 ②不能 ③不知道

D405b.[___]蚊虫叮咬会传播艾滋病吗？ ①会 ②不会 ③不知道

D405c.[___]与艾滋病病毒感染者或病人一起吃饭会感染艾滋病吗？ ①会 ②不会 ③不知道

D405d.[___]输入带有艾滋病病毒的血液会得艾滋病吗？ ①会 ②不会 ③不知道

D405e.[___]与艾滋病病毒感染者共用针具有可能得艾滋病吗？ ①可能 ②不可能 ③不知道

D405f.[___]感染艾滋病病毒的妇女生下的小孩有可能得艾滋病吗？ ①可能 ②不可能 ③不知道

D405g.[___]正确使用安全套可以减少艾滋病的传播吗？ ①可以 ②不可以 ③不知道

D405h.[___]只与一个性伴发生性行为可以减少艾滋病的传播吗？ ①可以 ②不可以 ③不知道

D406. [__] 你认为，目前艾滋病能预防吗？ 1)能 2)不能 3)不知道

D407. [__] 你认为，目前艾滋病能治好吗？ 1)能 2)不能 3)不知道

D408. 你知道到哪里去检测艾滋病病毒或艾滋病吗? ***(可多选,请在选项中划X)***

[__] D408a)疾控中心 [__]D408d)私人诊所 [__]D408b)公立医院

[__]D408e)社会民间组织 [__]D408c)乡镇卫生院 [__]D408f)其它 (请注明)_____________

D409.[__]最近一年，你曾经检测过艾滋病病毒或艾滋病吗？ 1)是 2)否 ***🡺跳至D411题***

D410.[__]HIV检测结果是? 1)阳性 2)阴性 3)不知道

D411.[__]如果你知道自己感染了艾滋病或艾滋病病毒，你会告诉别人吗？ 1)会 2)不会

D412. 你从哪里获得性病和艾滋病的知识的？***(可多选，不读出选项，请在选项中划X)***

[__]D412a)广播 [__]D412f)宣传栏、小册子、墙报等

[__]D412b)电视 [__]D412g)在当地开展的其他研究

[__]D412c)报纸、杂志、书籍 [__]D412h)戒毒所

[__]D412d)朋友 [__]D412i)红丝带(家园)

[__]D412e)当地卫生工作人员 [__]D412j)互联网

[__]D412k)其它________________

D413. [__] 你认为自己感染艾滋病的危险性有多大？

1)没有危险 2)低危险 3)中危险 4)高危险 5)不知道

**第五部分 性行为与吸毒行为情况**

E501. [__] 你第一次发生性行为时是多大岁数？ 1) [__|__]周岁 2)不记得

E502. [__] 你现在有爱人或女朋友吗？ 1)有 2)没有***跳至第E504题***

E503a. [__] 最近一年，你和固定性伴使用安全套的频率是：

1)总是***🡺跳至第E505题*** 2)大多数时间 3)大约一半时间 4)偶尔 5)从不

E503b. [__] 你和固定性伴不总是使用安全套的主要原因是什么？

1)自己不喜欢 2)性伴不喜欢 3)双方都不喜欢 4)相信对方没有性病 5)嫌麻烦

6)价格贵 7)当时身边没有 8)不知道到哪买 9)其它___________

E504. [__] 你最近一次与配偶或同居者发生性关系时使用安全套了吗？ 1)是 2)否

E505. [__]你以前还有其他不付费性伴吗？ 1)有 2)没有***🡺跳至第E508题***

E506. 过去1年，你共和多少人有过非婚性行为？

[__|__|__]个 ***(若填“0”， 🡺跳至E508题)***

E507. [__] 在非婚性行为中，你使用安全套的频率如何？

1)从来不用(0％) 2)偶尔(1％－25％) 3)大约一半(26％－74％)

4)绝大多数(75％－99％) 5)总是使用(100％)

E508. [__] 你周围的朋友(同事)有人找过小姐吗？ 1)有 2)没有 3)不知道

E509. 你第一次找小姐是什么时候？ [__|__|__|__]年[__|__]月

E510. [__]你第一次是因为什么原因找小姐的？

1)工作原因 2)跟朋友同事出去玩 3)生理需要 4)紧张、压力发泄 5)醉酒 6)其它________

E511. [__]一般来讲，你找小姐是自己一个人还是和朋友(同事)一起？

1)自己一个人 2)和朋友(同事)一起 3)都有过 4)其它________

E512. [__]一般来讲，你去哪里找小姐？

1)桑拿/洗浴中心 2)夜总会 3)卡拉OK厅/歌舞厅/酒吧 4)宾馆/酒店

5)越南街 6)其它（请注明）_______________

E513. 一般来讲，你每次付给小姐多少钱？ [__|__|__|__]元

E514. [__] 你一般喜欢找什么样的小姐？

1)熟人 2)新来的小姐 3)每次找不一样的 4)年轻漂亮的 5)其它________

E515. 找小姐的时候，你有没有做一些事情来判断小姐是否有性病？ 1)是 2)否

E516. [__]你曾有过男性性伴吗？ 1)有 2)没有

E517. [__]你曾经使用过毒品吗？ 1)是 2)否***🡺跳至F601题***

E518. [__]你曾经注射过毒品吗？ 1)是 2)否***🡺跳至F601题***

E519. [__]你共用过注射器吗？ 1)是 2)否***🡺跳至F601题***

E520. [__]你总共和多少人共用过注射器？ 1) [__|__|__]人 999)不知道

**第六部分:与付费性伴最近的性历史**

***最近1次的行为***

F601.你最近一次找小姐，付了多少钱？ [___|____|____|___] 元 9999)不知道

F602.[__]你最近一次与小姐发生性关系时，使用避孕套了吗？

1.用了: F602a.[__]由谁决定的？

1) 自己 2)小姐 3)共同决定

2.没用: F602b. [__]如果没有用避孕套，最主要的原因是什么？

1)没有安全套 2)对方不愿意使用 3)本人不愿意使用 4)已采用其它避孕方式

5)忘记使用 6)从不使用 7)其它（请注明）_____________________

F603.[__]你去的娱乐场所可以获得避孕套吗？ 1)可以 2)不可以 3)没有回答

F604.[__]你一般怎么获得避孕套的？(单选题)

1) 疾控中心免费提供 2) 自己购买 3)场所提供 4)其它(请注明)_______________

***最近1月的行为***

F605.你最近1个月，共找过多少个小姐? [__|__]个

99)不知道***🡺跳至第G701题***

F606.最近一个月，有几次你在与小姐发生性行为前喝过酒? [___|___]次 99)不知道

F607.最近一个月，有多少小姐在喝过酒后与你发生性行为? [___|___]个 99)不知道

F608.最近一个月和小姐发生性行为，你有几次是在射精前才戴上避孕套？

[___|___]次 99)不知道

F609.最近一个月和小姐发生性行为，你有几次在性交过程中自己摘掉避孕套？

[___|___]次 99)不知道

F610. [__] 最近一个月，你和商业性性伴使用安全套的频率是：

1)总是 2)大多数时间 3)大约一半时间 4)偶尔 5)从不

**第七部分: 最近1个付费性伴（小姐）的信息**

***G701．性伴信息***

G701a) [__]国籍 1)中国 2)越南 3)其它(请注明)____________

G701b) 年龄 ______周岁

G701c) [__]是否使用毒品？ 1)是 2)否 3)不知道

G701d) [__]是否使用避孕套？ 1)是 2)否 3)不知道

G701e) [__]性交前是否喝酒？ 1)是 2)否 3)不知道

------------------------ 调查结束-------------------------
