# Supplementary material for: HIV prevalence and correlated factors among male clients of female sex workers in a border region of China
Source: PLoS One. 2019 Nov 7;14(11):e0225072. doi: 10.1371/journal.pone.0225072 (PMC6837524; doi:10.1371/journal.pone.0225072)
Supplement: S5 Appendix — (DOC) [file pone.0225072.s005.doc]

**Health behaviors questionnaire for male clients of FSWs**

PID No.**□□□□□□**

Hello，This is an investigation designed by China center for disease control and prevention, in order to understand your attitude to disease and health problems. Your name will not appear on the questionnaire and each response to the questions will be protected as confidential information. If you have any question please ask us at any time. Your truthfully response to the questions will help us get better understanding to disease and health problems among male clients of FSWs. Thank you for your cooperation.

Filling explanation：Please fill numbers, codes or “X” in the “[__]”, multiple choice questions please fill “X” in the “[__]” in front of optional answers, please write down Chinese on the “______”

Investigator____________Date：____________； Verifier_____________Date_____________

**Section 1 Basic Information**

A101.Date of birth [__|__|__|__]Year [__|__]Month

A102.[__]Marital status 1)Single 2)Married 3)cohabiting 4)Divorced or widowed

A103.[__]Registered residence

1)Hekou county 2)Other region in Honghe Prefecture 3)Other region in Yunnan Province(Except Honghe)

4)Other Province in China（______） 5)Foreign country（______）***🡺Jump to A104b***

A104a.[__]Choose ethnicity if Chinese ***🡺Jump to 105 after answer this question***

1)Han 2)Hui 3)Hani 4)Yi 5)Other__________

A104b.[__]Choose ethnicity if Vietnamese

1)Kinh 2)Lu 3)Hoa 4)Thai 5)Ha Nhi 6)Tay 7)Khmer 8)Other__________

A105.[__]Job 1)Merchant 2)Worker 3)Driver 4)Farmer 5)Other____________________

A106.[__]Education 1)No schooling 2)Primary school 3)Middle school 4)High school 5)College or university

A107.[__]Family address 1)Village 2)County 3)Middle or small city 4)Big city

A108.[__]Can you understand Vietnamese？ 1）Yes 2）Partly 3）No

A109.[__]Can you speak Vietnamese？ 1）Yes 2）Partly 3）No

**Section 2 Living information**

B201.Why did you come to Hekou county？(Multiple choice)

[__]B201 1)Busniess trip [__]B201 2)For family [__]B201 3)For job

[__]B201 4)For tourism [__]B201 5)Local resident [__]B201 6)Other__________

B202.Change residence in last 5 years (to other county or city) [__|__|__]Times ***If 0🡺Jump to B204***

B203.Why did you change residence？***(Multiple choice)***

B203a). [__]Busniess trip B203b) .[__]For family B203c) .[__]For job

B203d).[__]In order to earn more money B203e) .[__]Other(Please note)__________________

B204.[__]Currently monthly income（CNY） 1)0

2)1～ 3)500～ 4)1000～ 5)2000～ 6)3000～ 7)5000～

B205.[__]Family monthly income（CNY） 1)0

2)1～ 3)1000～ 4)2000～ 5)4000～ 6)6000～ 7)8000～

B206.[__]How many children do you have? 1) 0 2) 1 3) 2 4) 3 5) > 3

B207.[__]Do you have a habit of drinking？ 1)Yes 2)No

B208.[__]Do you have a habit of smoking? 1)Yes 2)No

**Section 3 Medical experiences**

C301.Do you have the following experiences？ 1)Yes 2)No

C301a). [__] Tooth extraction/Filling tooth C301b). [__]Blood or other blood products transfusions

C301c). [__] Blood donation C301d). [__] Tattoo

C301e). [__] Circumcision C301f). [__] Surgical operation

C302.Have you had the following symptoms in the past 12 months？***(Multiple choice )*** 1)Yes 2)No

C302a) [__] Genital itching C302b) [__] Urethral pain

C302c) [__] Suppurated or secreta near the [urethral](javascript:;) [orifice](javascript:;) C302d) [__] [Hyperplasia](javascript:;) or ulceration in gentitals

C302e) [__] [huckle](javascript:;) [lymphadenectasis](javascript:;) C302f) [__] None of the above ***🡺Jump to D401***

C303. Do these symptoms you still have？ 1)Yes 2)No

C303a) [__] Genital itching C303b) [__] Urethral pain

C303c) [__] Suppurated or secreta near the [urethral](javascript:;) [orifice](javascript:;) C303d) [__] [Hyperplasia](javascript:;) or ulceration in gentitals

C303e*)* [__] [huckle](javascript:;) [lymphadenectasis](javascript:;)

**Section 4 Consciously risk and related knowledge of HIV/STDs**

D401. [__] Do you know sexual transmitted diseases (STDs)? 1)Yes 2)No***🡺Jump to D405***

D402. [__] How do you think your risk level of acquiring STDs？

1)No risk 2)Low risk 3)Middle risk 4)High risk 5)Don’t know

D403. [___] Have you been diagnosed with STDs in the last year？ 1)Yes 2)No***🡺Jump to D405a***

D404. Which STDs have you been diagnosed with？(Multiple choice)

[__]D404a).Gonorrhoea [__]D404b).Syphilis [__]D404c. Chlamydia trachomatis

[__]D404d. [Condyloma](../../../../D:/%25E6%259C%2589%25E9%2581%2593/Dict/6.3.69.8341/resultui/frame/javascript:void(0)%3B) [acuminata](../../../../D:/%25E6%259C%2589%25E9%2581%2593/Dict/6.3.69.8341/resultui/frame/javascript:void(0)%3B) [__]D404e).[Herpes](../../../../D:/%25E6%259C%2589%25E9%2581%2593/Dict/6.3.69.8341/resultui/frame/javascript:void(0)%3B) [progenitalis](../../../../D:/%25E6%259C%2589%25E9%2581%2593/Dict/6.3.69.8341/resultui/frame/javascript:void(0)%3B)

[__]D404f).Other (Please note)_______________

D405a.[___]Can people infected HIV be identified through appearance? ①Yes ②No ③Don’t know

D405b.[___]Can HIV/AIDS spread through mosquito bites? ①Yes ②No ③Don’t know

D405c.[___]Does it increase the risk of acquiring HIV if you dine

with HIV infected people or AIDS patients? ①Yes ②No ③Don’t know

D405d.[___]Can people infect HIV due to transfuse Blood that contaminated with HIV?

①Yes ②No ③Don’t know

D405e.[___]Does needle sharing with HIV infected people increase the risk of acquiring HIV?

①Yes ②No ③Don’t know

D405f.[___]May the children of HIV infected women also infected with HIV?

①Yes ②No ③Don’t know

D405g.[___]Can condom use reduce HIV spread through sexual transmission？

①Yes ②No ③Don’t know

D405h.[___]Can keep one regular sexual partner reduce the risk of acquiring HIV?

①Yes ②No ③Don’t know

D406. [__]Do you think HIV transmission can be prevented？ 1)Yes 2)No 3)Don’t know

D407. [__]Do you think HIV transmission can be cured？ 1)Yes 2)No 3)Don’t know

D408. Do you know where to test HIV? ***(Multiple choice)***

[__] D408a)Center for disease control and prevention [__]D408d)Private clinics

[__]D408b)Hospitals [__]D408e)Non-governmental organizations [__]D408c)Township health center

[__]D408f)Other(Please note)_____________

D409.[__]Have you tested HIV in the last year？ 1)Yes 2)No ***🡺Jump to D411***

D410.[__]The result of HIV testing? 1)Positive 2)Negative 3)Don’t know

D411.[__]Will you inform others if you acquired HIV？ 1)Yes 2)No

D412. Where did you get the HIV related knowledge from？***(Multiple Choice)***

[__]D412a)Broadcasting [__]D412f)Brochures of HIV prevention

[__]D412b)TV [__]D412g)Local scientific research

[__]D412c)News paper/book/journal [__]D412h)Police

[__]D412d)Friends [__]D412i)Non-governmental organizations

[__]D412e)Local health officials [__]D412j)Internet

[__]D412k)Other________________

D413. [__]How do you think your risk level of acquiring HIV？

1)No risk 2)Low risk 3)Middle risk 4)High risk 5)Don’t know

**Section 5 Sexual and drug using behaviors**

E501. [__] How old are you at your sexual debut？ 1) [__|__]Years old 2)Can’t remember

E502. [__] Do you have wife or girl friend now？ 1)Yes 2)No ***Jump to E505***

E503a. [__] The frequency of condom use with your regular sexual partner：

1)Always***🡺Jump to E505*** 2)Most time 3)Half the time 4)Occasionally 5)Never

E503b. [__] Why don’t you keep using condom with your regular sexual partner？

1)Don’t want to use by myself 2) Don’t want to use by regular sexual partner 3)Trust each other

4)Forgot to use 5)Don’t have condom then 6)Other___________

E504. [__]Did you use condom with your regular sexual partner in the last episode of sexual behavior？

1)Yes 2)No

E505. [__]Do you have other no-paid sexual partners？ 1)Yes 2)No ***🡺Jump to E508***

E506. How many no-paid sexual partners had sexual behaviors with you the last year？

[__|__|__] ***(If “0”，🡺jump to E508)***

E507. [__] The frequency of condom use with no-paid sexual partner？

1)Never(0％) 2)Occasionally(1％－25％) 3)Half the time(26％－74％)

4)Most time(75％－99％) 5)Always(100％)

E508. [__] Have your friends or colleagues ever had commercial sexual behavior？ 1)Yes 2)No 3)Don’t know

E509. Date of commercial sexual debut？ [__|__|__|__]Year[__|__]Month

E510. [__]The reason of your debut in commercial sex

1)Due to work 2)Due to friends or colleagues 3)Physiological need 4)Release pressure

5)Drunk 6)Other________

E511. [__]Do you always meet FSWs alone or with friends/colleagues？

1) Alone 2)With friends or colleagues 3)Both 1 and 2 4)Other________

E512. [__]Which is the primary place for you to meet FSWs？

1) Bath center 2)Night club 3)Karaoke/Dance hall/Pub 4)Hotel

5) Vietnamese street 6)Other（Please note）_______________

E513. In general, how much you pay for commercial sex？ [__|__|__|__]CNY

E514. [__] Which genre of female sex workers do you like most？

1) Acquaintance 2)Newly coming 3)Different at each time 4)Young and beauty 5)Other________

E515. Do you always do something to estimate whether FSWs infected STDs？ 1)Yes 2)No

E516. [__]Have you ever had male sexual partners？ 1)Yes 2)No

E517. [__]Have you ever used drugs？ 1)Yes 2)No***🡺Jump to F601***

E518. [__]Have you ever injected drug？ 1)Yes 2)No***🡺Jump to F601***

E519. [__]Have you ever shared needles？ 1)Yes 2)No***🡺Jump to F601***

E520. [__]How many people shared needles with you？ 1) [__|__|__] 999)Forgot

**Section 6 Current commercial sexual characteristics**

***The last episode of commercial sexual behavior***

F601.How much do you paid for the last commercial sex？ [___|____|____|___] CNY 9999)Don’t know

F602.[__]Did you use condom in the last episode of commercial sex？

1.Yes: F602a.[__]Who decided？

1) Myself 2)Female sex worker 3)Both 1 and 2

2.No : F602b. [__]The reason why not used condom？

1)Didn’t have condom 2)Refused to use by FSW 3) Refused to use by myself

4)Other contraceptive measures have been taken 5)Forgot to use 6)Never use

7)Other（Please note）_____________________

F603.[__]Can it be convenience to get condoms in the brothel based entertainment venues？ 1)Yes 2)No

F604.[__]The main source of condoms？

1) Provide free for charge by CDC 2) Purchase by myself 3)Provide by FSWs

4)Other(Please note)_______________

***Commercial sexual behavior in the last month***

F605.How many FSWs had sexual behaviors with you in the last month? [__|__]

99)Forgot***🡺Jump to G701***

F606.How many times you drunk before commercial sexual behaviors last month?

[___|___] 99)Don’t know

F607.How many FSWs had commercial sexual behavior with you after drunk last month

[___|___] 99)Don’t know

F608.How many times you put on condom just before ejaculation last month？

[___|___] 99)Don’t know

F609.How many times you take down condom during commercial sexual behaviors last month？

[___|___] 99)Don’t know

F610. [__] The frequency of condom use with commercial sexual partners：

1)Always 2)Most time 3)Half the time 4)Occasionally 5)Never

**Section 7 Information of the last commercial sexual partner**

***G701．Information of the last commercial sexual partner***

G701a) [__]Nationality 1)China 2)Vietnam 3)Other(Please note)____________

G701b) Age ______Years old

G701c) [__]Does she had drug using behavior？ 1)Yes 2)No 3)Don’t know

G701d) [__]Used condom？ 1)Yes 2)No 3)Don’t know

G701e) [__]Drinking before sexual behavior？ 1)Yes 2)No 3)Don’t know

------------------------ Investigation Finished -------------------------
